# Smart Watch Sensors for Tremor Assessment in Parkinson’s Disease—Algorithm Development and Measurement Properties Analysis

**DOI:** 10.3390/s25144313

**Published:** 2025-07-10

**Authors:** Giulia Palermo Schifino, Maira Jaqueline da Cunha, Ritchele Redivo Marchese, Vinicius Mabília, Luis Henrique Amoedo Vian, Francisca dos Santos Pereira, Veronica Cimolin, Aline Souza Pagnussat

**Affiliations:** 1 Movement Analysis and Rehabilitation Laboratory, Universidade Federal de Ciências da Saúde de Porto Alegre (UFCSPA), Porto Alegre 90050-170, Brazil; giulias@ufcspa.edu.br (G.P.S.); mairaj@ufcspa.edu.br (M.J.d.C.); ritchele@ufcspa.edu.br (R.R.M.); franciscap@ufcspa.edu.br (F.d.S.P.); 2 Rehabilitation Sciences Graduate Program, Universidade Federal de Ciências da Saúde de Porto Alegre (UFCSPA), Porto Alegre 90050-170, Brazil; 3 Department of Physical Therapy, Universidade Federal de Ciências da Saúde de Porto Alegre (UFCSPA), Porto Alegre 90050-170, Brazil; 4 Department of Electronics, Information and Bioengineering, Politecnico di Milano, 20133 Milan, Italy; veronica.cimolin@polimi.it; 5 IRCCS Istituto Auxologico Italiano, 28824 Piancavallo, Italy; 6 Health Sciences Graduate Program, Universidade Federal de Ciências da Saúde de Porto Alegre (UFCSPA), Porto Alegre 90050-170, Brazil

**Keywords:** Parkinson’s disease, tremor, smartwatch, wearable sensors, inertial measurement unit, spectral analysis, upper limb, movement disorder

## Abstract

**Highlights:**

**What are the main findings?**

- A smartwatch-based algorithm was developed to assess upper limb tremor in Parkinson’s Disease (PD) using spectral and spatiotemporal features.
- The algorithm showed moderate to strong agreement with a commercial IMU and was capable of distinguishing PD patients from healthy individuals.

**What are the implications of the main findings?**

- Smartwatches can be used as low-cost and accessible tools for remote clinical assessment of PD motor symptoms.
- This wearable approach may support the transition of tremor evaluation from controlled lab environments to free-living settings.

**Abstract:**

Parkinson’s disease (PD) is a neurodegenerative disorder commonly marked by upper limb tremors that interfere with daily activities. Wearable devices, such as smartwatches, represent a promising solution for continuous and objective monitoring in PD. This study aimed to develop and validate a tremor-detection algorithm using smartwatch sensors. Data were collected from 21 individuals with PD and 27 healthy controls using both a commercial inertial measurement unit (G-Sensor, BTS Bioengineering, Italy) and a smartwatch (Apple Watch Series 3). Participants performed standardized arm movements while sensor signals were synchronized and processed to extract relevant features. Statistical analyses assessed discriminant and concurrent validity, reliability, and accuracy. The algorithm demonstrated moderate to strong correlations between smartwatch and commercial IMU data, effectively distinguishing individuals with PD from healthy controls showing associations with clinical measures, such as the MDS-UPDRS III. Reliability analysis demonstrated agreement between repeated measurements, although a proportional bias was noted. Power spectral density (PSD) analysis of accelerometer and gyroscope data along the *x*-axis successfully detected the presence of tremors. These findings support the use of smartwatches as a tool for detecting tremors in PD. However, further studies involving larger and more clinically impaired samples are needed to confirm the robustness and generalizability of these results.

## 1. Introduction

Parkinson’s Disease (PD) is a neurodegenerative disorder that affects approximately 1% of the global population over the age of 65 and is recognized for its widespread impact on motor functions, quality of life, and daily activities [1,2]. Tremors in the upper limbs are commonly observed in individuals with PD. They can significantly impair fine motor skills and the ability to carry out daily tasks, such as feeding, dressing, and other routine tasks [3,4,5]. These motor deficits can substantially diminish the quality of life, work capabilities, and social interactions of affected individuals, underscoring the significant impact of the disease [6]. Tremor, one of the most visible symptoms in the early stages of PD, may contribute to social isolation due to the stigma often associated with the condition [1,2].

The evaluation of tremors in PD is of great importance. This assessment can take various forms, ranging from the implementation of comprehensive scales specifically designed for PD, such as the Unified Parkinson’s Disease Rating Scale motor examination section (UPDRS III) [7] and the Clinical Rating Scale for Tremor [8], to the utilization of broader upper limb assessment tools such as the Nine-Hole Peg Test [8] or the Box and Blocks Test [9], among others [8]. Although these types of assessments are widely used, they all have some limitations. These range from low specificity in tremor evaluation, as seen in dexterity tests like the Nine-Hole Peg Test and others, to clinical scales that, despite being easy and cost-effective, require time for practical application and are highly dependent on the evaluator [8]. Furthermore, all these tests capture only a specific moment during which the observer is assessing the patient, and therefore may fail to reflect the reality of the patient’s daily life, where tremors may be influenced by factors such as stress and task complexity. Thus, there is a clear need for monitoring solutions that are more efficient, tremor-specific, continuous, patient-friendly, and wearable.

In this context, wearable sensors have emerged as a solution, offering a quantitative, objective and trustworthy method of analysis. Therefore, wearable sensors have been increasingly employed in numerous studies, providing a robust approach for generating accurate findings. Some investigations utilize electromyography to assess upper limb tremors in individuals with PD [10,11] while others leverage technologies such as accelerometers and gyroscopes to quantify arm movements in these patients, assessing tremor measures such as frequency, amplitude, and spectral characteristics of the signal [12,13,14,15,16,17,18,19]. While the utilization of wearable devices such as electromyographs, gyroscopes, and accelerometers has emerged as a practical and reliable technique for evaluating upper limb movement in PD, their application remains largely confined to laboratory and academic settings. This limitation arises from the reliance on costly and scarcely available devices, along with their complex handling and operational demands.

Within this context, the use of sensors embedded in smartphones and smartwatches for the clinical assessment of symptoms—particularly those related to upper limb movement in PD—emerges as a promising and practical tool [20,21,22]. This approach not only assists clinicians in quantifying and tailoring treatment plans, whether pharmacological or physiotherapeutic, but also facilitates early symptom detection, enabling timely intervention and improved disease management.

Indeed, smartwatches are increasingly used to assess health metrics such as fall risk in older adults and, more recently, to monitor tremors in PD. Equipped with gyroscopes, some devices track tremor intensity and frequency, while others aim to reduce tremor severity. Patients can also manually record aspects of their well-being—for example, after taking medication—with data uploaded to the cloud for remote access by both patients and healthcare providers. However, there remains a lack of clarity regarding the validity and reliability of these devices, as few have undergone formal validation or received approval for clinical use [23].

Given this context, this study aimed to develop an algorithm for detecting tremors in individuals with PD using smartwatch sensors and to evaluate its measurement properties. The proposed algorithm, developed by the authors, was specifically tailored to the characteristics of smartwatch sensors, incorporating a customized signal processing and feature extraction pipeline suitable for tremor detection in PD. First, we assessed the agreement between measurements obtained using smartwatch sensors and a commercial wearable inertial sensor. Second, we examined the algorithm’s discriminant validity, concurrent validity, reliability, and accuracy. By exploring this innovative approach, we aim to contribute to the enhancement of clinical care and the quality of life for individuals living with PD.

## 2. Materials and Methods

### 2.1. Study Design

This observational cross-sectional study aimed to develop an algorithm for detecting tremors in individuals with PD and to investigate its measurement properties (discriminant validity, concurrent validity, reliability, and accuracy). The study was conducted at the Universidade Federal de Ciências da Saúde de Porto Alegre (UFCSPA), Porto Alegre, Brazil. We adhered the STROBE (Strengthening the reporting of observational studies in epidemiology) guidelines for cross-sectional studies [24] and the STARD (Standards for Reporting of Diagnostic Accuracy Studies reporting guidelines) [25]. The study was approved by the Ethics and Research Committee of UFCSPA (CAAE 30407320.9.0000.5345). Written informed consent was obtained from all participants prior to the commencement of the procedures.

### 2.2. Participants

Participants were recruited for convenience through a flyer posted on social media and were selected based on the following eligibility criteria: (1) clinical diagnosis of Parkinson’s Disease (PD) according to the London Brain Bank Criteria (2006) [26]; (2) self-reported presence of tremor; (3) age between 20 and 90 years; and (4) both male and female participants were eligible. In addition, a control group of healthy individuals matched by gender was included as a reference. Exclusion criteria comprised the presence of cognitive impairment, defined as a score below 21 on the Mini-Mental State Examination (MMSE) for illiterate individuals, or below 25 for literate individuals [27].

### 2.3. Instruments

**Figure 1:**
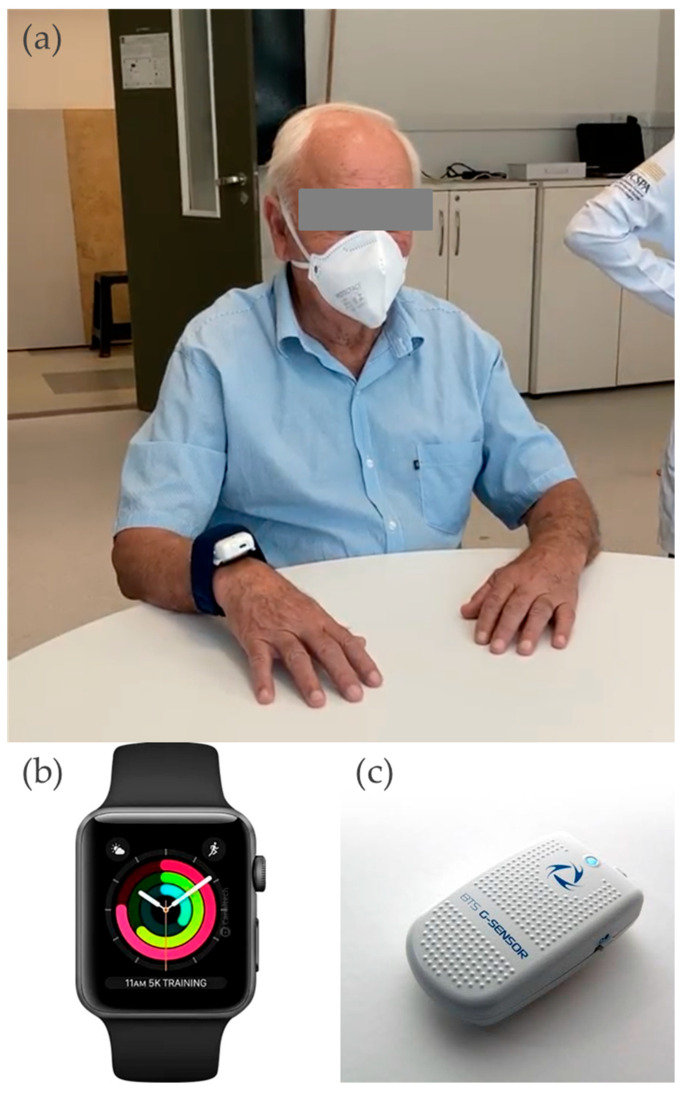
Data Acquisition Set-up and Instruments. Note: (**a**) Data Acquisition Set-up (**b**) Smart Watch (**c**) Commercial IMU (BTS Bioengineering, Milan, Italy).

The G-Sensor (BTS G-Walk, BTS Bioengineering Corporation, Garbagnate Milanes, Italy) used in this study is a commercial Inertial Measurement Unit (IMU) composed of a three-axis accelerometer, three -axis gyroscope, and three -axis magnetometer (Figure 1c). It is widely adopted in laboratory settings due to its high reliability and the availability of dedicated software (BTS G-STUDIO, version 2.6.12.0), which enables wireless activation via Bluetooth, real-time visualization of sensor orientation and signal, and selective export of raw data. The accelerometer and gyroscope signals were sampled at 100 Hz.

As a comparative and accessible tool, we employed the Apple Watch Series 3 as the smartwatch sensor. It was developed by Apple (Apple Inc., Cupertino, CA USA), it is equipped with a three-axis accelerometer and a three-axis gyroscope. This device was selected for its low cost, intuitive user interface, and broad availability in the consumer market, making it a practical option for clinical and home use. To access the raw inertial data from the smartwatch, a specific iOS application (SensorLog v53, Stuttgart, Germany) [28] was manually configured through the touchscreen display at the start of each recording session application. SensorLog is a free and widely available tool from the Apple App Store that has been validated in multiple research studies. It allows users to record and export accelerometer and gyroscope data with user-defined settings, and transmit them to a master iPhone that also has the same app installed and is connected to the Apple Watch. The declared sampling frequency of the smartwatch was 100 Hz.

This combination of a gold-standard laboratory-grade IMU and a commercially available smartwatch aligned with the goal of promoting an accessible assessment method—not only for clinicians in formal care settings but also for individuals with PD in home environments, particularly in resource-constrained contexts.

### 2.4. Procedures

Following the consent process, patients underwent a comprehensive clinical assessment that included inquiries into demographic attributes such as weight, height, and conditions like hypertension and diabetes. Subsequently, subjects were administered the Montreal Cognitive Assessment (MoCA) questionnaire to evaluate their cognitive status. For individuals diagnosed with PD, the Unified Parkinson’s Disease Rating Scale motor examination section (UPDRS III) [7] was conducted to assess motor impairments, and the Hoehn and Yahr disease staging [28] evaluation was employed to determine the stage of disease progression [29].

#### 2.4.1. Arm Movement Acquisition

The acquisition of arm movement data commenced with subjects seated in a chair facing a table at elbow level. Both devices were concurrently positioned on the subject’s arm using an elastic band that fixed them together (with the G-sensor placed vertically above the smartwatch) (Figure 1a). Upon device activation, participants were instructed to perform a high-speed, wide-amplitude shoulder flexion movement in response to an auditory cue. This initial movement facilitated subsequent data synchronization. Immediately following this, participants were asked to reach for a target situated at 80% of their arm’s length; this movement was repeated three times consecutively. Once the movement sequence was completed, the sensors were deactivated, and data collection concluded. PD subjects performed the movement with their most affected side, while control subjects used their dominant hand [30].

The chosen upper limb activity, the “reach” movement, is a functional action commonly used in daily activities, such as reaching for a glass to drink water, feeding oneself, or grasping an object in front of oneself. We opted to repeat the movement three times for each acquisition. This decision was made because repetitive and cyclic movements tend to evoke PD symptoms more effectively, thus facilitating symptom manifestation [31,32]. This approach aligns with previous studies that have quantitatively assessed arm movement in individuals with PD through repeated and cyclic movements, including finger tapping, finger-to-nose movements, forearm pronation-supination, and opening and closing the hand [21,33,34,35,36].

#### 2.4.2. Data Analysis

- Signals Pre-processing

The proposed algorithm, developed by the authors, was specifically adapted to the characteristics of smartwatch sensors, incorporating a customized signal processing and feature extraction pipeline suitable for tremor detection in PD. The raw data were exported from the devices (IMU and smartwatch) and underwent a preprocessing procedure to filter out possible noise and select the specific signal frequencies where tremors occur in PD. This process is detailed below. Data analysis was performed using MATLAB (R2022a, MathWorks Inc., Natick, MA, USA). During the file import process, the headers in the CSV files related to smartwatch data were manually reassigned to the first row because they were randomly placed in the files for unknown reasons. Additionally, timestamps and corresponding variables that were repeated twice or more were removed from these files.

The algorithm implemented in this study is rule-based and does not rely on machine learning or supervised training procedures. It was constructed using predefined signal characteristics obtained from the literature and was refined through pilot testing. Parameters such as amplitude thresholds, frequency ranges, and temporal windows were empirically selected to best capture parkinsonian tremor patterns. Consequently, there was no need for a separate training and validation dataset, and the validation was carried out using the same recordings obtained during the experimental protocol, through comparisons across devices and participants.

The declared sampling frequency of the smartwatch and IMU was reported as 100 Hz. However, calculations based on the timestamp and number of samples revealed that the actual frequency was not precise, necessitating resampling. Upon plotting the raw data, it became evident that the IMU and the smartwatch utilized different axial coordinate systems. Consequently, a transformation was necessary to standardize the coordinate systems. Specifically, the accelerometer’s *z*-axis and the gyroscope’s *x* and *y*-axes were multiplied by −1 to effect the required change.

To ensure the elimination of noise and preservation of valuable information, the raw data was filtered. Given that human movements typically exhibit frequencies below 20 Hz, a Butterworth low-pass filter was chosen for this purpose. The filter was designed with a cutoff frequency of 20 Hz and was of the 4th order.

The frequency of human movement refers to the rate at which the body undergoes motion or changes in position. In the context of signal processing, it is crucial to consider the frequency range of interest when selecting an appropriate filter. For human movements, frequencies below 20 Hz are typically relevant, as they capture the dynamic nature of various activities. The order of a Butterworth filter determines its steepness of attenuation in the stopband. A higher order signifies a more rapid transition between the passband and the stopband, resulting in increased suppression of frequencies beyond the cutoff point. In this case, a 4th-order Butterworth filter was chosen to effectively attenuate frequencies above the desired cutoff frequency of 20 Hz, thereby ensuring the removal of higher-frequency noise components while retaining the essential information related to human movements.

All variables were standardized to make them comparable and eliminate the effects of scale and magnitude. The standardization process involved subtracting the mean of the variable from each data point and then dividing the result by the standard deviation. This transformation resulted in a variable with a mean of zero and a standard deviation of one. Mathematically, the standardization of a variable “x” can be represented as:

z = (x − μ)/σ(1)

where z represents the standardized value, x is the original value of the variable, μ is the mean, and σ is the standard deviation.

Standardization is advantageous in data analysis because it enables comparisons between variables with different units and scales. By standardizing variables, they can be directly compared based on their relative positions within the distribution. This process is particularly useful when dealing with variables with two different scale systems. Additionally, standardization aids in identifying outliers, as they often appear as extreme values beyond a certain threshold in the standardized scale. It also facilitates the interpretation of variables on a common scale, allowing for meaningful comparisons of their effects. Overall, standardization is a fundamental step in data preprocessing, providing a way to transform variables into a standardized format that simplifies analysis and enhances their comparability.

Subsequently, the data were also normalized. Normalization is a data transformation technique that adjusts the values of variables to a specific range. For this application, the chosen range was between −1 and 1, where negative values represent deceleration and positive values represent acceleration, while preserving the relative relationships between the data points.

The process of normalizing data involves scaling the values of each variable to fit within this specified range. The method used is min-max normalization, where the minimum value of the variable is mapped to −1, and the maximum value is mapped to 1. The other values in between are linearly scaled accordingly. Mathematically, the normalization of a variable “x” can be represented as:

x normalized = (x − min(x))/(max(x) − min(x))(2)

where x normalized is the normalized value of x, min(x) is the minimum value of x, and max(x) is the maximum value of x.

In the present study, the alignment procedure was based on a deliberate and standardized initial up/down shoulder flexion movement, where the sensors were attached at the wrist. This movement was performed rapidly and with a large amplitude. It was chosen because it is easily distinguishable from the specific upper-limb task under analysis, providing a clear reference point for temporal alignment. Although this movement was performed only once, it was considered sufficiently distinct from the specific movements under investigation, thus providing a potential reference point. By employing this approach, spikes were observed at the beginning of each signal recording, exhibiting a slight resemblance in both measurement systems.

However, relying solely on this initial peak was deemed inadequate due to the presence of considerable delay in the signals and the inability to consistently utilize it as a reliable trigger for all subjects’ recordings. The signal processing approach employed in this study involved using the built-in MATLAB function “findpeaks” to locate the positions of peaks in both signals. To effectively capture the desired peaks, specific criteria such as prominence and distance thresholds were set. Next, the signals were flipped, and the same “findpeaks” function was applied to identify the locations of valleys. The objective was to determine the first and last peaks (or valleys) present in the signals, representing the beginning and end of the recordings.

This process was repeated for each variable detected by the two sensors. Additionally, the length of the signal was measured from the first peak to the final one, assuming that both sensors have the same sampling frequency and, consequently, similar signal lengths. Subsequently, the difference between the IMU’s signal and the smartwatch’s signal was calculated. The variable exhibiting the minimum difference was selected, and this value was utilized as a delay to align all the variable signals. This alignment ensures that corresponding events in the signals occurred simultaneously, facilitating further analysis and comparison.

By applying this systematic approach, the study aimed to synchronize and align the variable signals obtained from the IMU and smartwatch sensors, enabling accurate and reliable data analysis while mitigating any potential temporal discrepancies between the measurements.

Sensor calibration followed manufacturer protocols and was verified via static and dynamic reference recordings prior to data collection.

- Features Calculation

##### Spatio-Temporal Analysis

For each variable we calculated the mean and the standard deviation.

##### Spectral Analysis

In the frequency domain, the power spectral density (PSD) was calculated to examine the frequency content or power distribution in a signal. The PSD provides valuable information about the strength or intensity of various frequency components present in the signal. It is calculated by taking the Fourier Transform of a signal and then calculating the squared magnitude of the resulting complex spectrum. This squared magnitude represents the power at each frequency component of the signal. The PSD is typically expressed in W/Hz.

The PSD value at a specific frequency indicates the amount of power or energy contained within that frequency range. Higher PSD values at a given frequency denote higher power or energy, whereas lower values denote lower power or energy. For this application, the PSD was calculated in the frequency range of 3.5–7 Hz because this is the range where Parkinson’s tremors may arise [12,37].

In particular, the ratio between the integral of the PSD curve in the range of 0–20 Hz, which is considered the range where human movement occurs, and the integral in the range of 3.5–7 Hz, which is the range where Parkinson’s tremors occur, was calculated. Higher values of this ratio could indicate the possible presence of pathological tremors [38].

#### 2.4.3. PD Symptom Presence

During the arm movement, a video recording was conducted. Subsequently, a licensed physiotherapist experienced in scoring the MDS-UPDRS III scoring, who was blinded to outcomes such as the Hoehn and Yahr stage, time since diagnosis, and MDS-UPDRS III score, assessed the presence or absence of symptoms such as resting tremors and action tremors. This clinical analysis of the movement was repeated for each acquisition and served as the gold standard for evaluating the test’s performance in detecting symptoms of Parkinson’s Disease.

#### 2.4.4. Nine-Hole Peg Test

The Nine-Hole Peg Test was conducted to compare the obtained data with a functional assessment measure of upper limb movement. This test measures the time that it takes to remove small pegs from a concave receptacle, insert them into a series of holes, and then return them to the receptacle. Participants performed three timed trials using their more affected hand if they were in the PD group and their dominant hand if they were in the control group. A mean score was obtained from these three measures [39,40].

#### 2.4.5. Algorithmic Assessment Features

The measurement properties of the algorithm and test encompassed various aspects. Firstly, the agreement between measures obtained from both the smartwatch and a commercially available IMU was assessed using the Bland-Altman analysis. Following this, discriminative validity was evaluated by assessing the algorithm’s ability to differentiate between individuals with and without PD based on smartwatch data. Concurrent validity, gauging the algorithm’s capability to evaluate the proposed construct, was examined through its correlation with the clinical measurement variable MDS-UPDRS. The analysis of reliability, focusing on the consistency of measures over time, involved comparing two consecutively acquired measurements. Lastly, accuracy measures were determined through sensitivity and specificity analyses of the test’s ability to detect both resting and action tremors and differentiate between individuals with and without PD.

### 2.5. Statistical Analysis

The sample size was determined using the G-Power 3.1.9.7 software based on a previous study [38], considering an effect size of 0.70, 80% power and an alpha value of 0.05 to detect a minimum clinical difference of 10% in Power Spectral Density measured by the smartwatch unity’s gyroscope on the *x* axis (Spectral power, angular velocity on x axis). Twenty-six participants on each group were estimated as necessary for this study.

Demographic data were described by means and standard deviations, and frequencies. Shapiro–Wilk tests were used to evaluate the normality of the continuous variables.

A Bland–Altman analysis was performed to verify the agreement between G-sensor and Smart Watch measures. The differences and the mean were calculated for all features via the following formulas:

DIF − cIMU − SW(3)

μ = (cIMU + SW)/2(4)

where DIF is the difference between measures, cIMU refers to the commercial IMU, SW refers to the Smart Watch and µ is the mean between measures.

After the graphs were created, a one-sample T-test was performed using the differences between measures to verify whether they were significantly higher than zero. Additionally, a linear regression was conducted using the differences and means between measures to check for the existence of proportional bias across the differences. If the regression was statistically significant, it would indicate that the differences between measures tended to concentrate either above or below the mean of differences.

Correlations between the commercial IMU and smartwatch data were calculated using Pearson or Spearman correlations for parametric and non-parametric data, respectively. Discriminative validity, assessed by the differences in upper limb movement between the PD and control groups, was verified through the Mann–Whitney test for non-parametric variables and a T-test for parametric variables.

Concurrent validity, assessed by correlations between smartwatch features and MDS-UPDRS, Nine-Hole Peg test, and time since diagnosis data, was calculated using Pearson or Spearman correlations for parametric and non-parametric data, respectively.

Reliability, assessed by the agreement between the first and second measures (M1 and M2, respectively), was performed using Bland-Altman analysis [41,42]. The differences and the mean between measures were calculated for all features using the following formulas:

DIF = M1 − M2(5)

μ = (M1 + M2)/2(6)

where DIF is the difference between measures, M1 is the first acquisition, M2 is the second acquisition and µ is the mean between measures.

After the graphs were created, a one-sample T-test was performed using the differences between measures to verify whether they were significantly higher than zero. Additionally, a linear regression was conducted using the differences and means between measures to check for the existence of proportional bias across the differences. If the regression was statistically significant, it would indicate that the differences between measures tended to concentrate either above or below the mean of the differences.

For the accuracy evaluation, the sensitivity and specificity of the test were assessed through ROC curve analysis. After determining the area under the curve (AUC), an optimal cutoff point was identified to optimize the sensitivity and specificity for each evaluated measure. The test accuracy in assessing the variables was considered significant when the AUC exceeded 0.7 [43].

## 3. Results

Twenty-one individuals with Parkinson’s Disease (PD) and twenty-seven healthy controls were included. The demographic data and clinical characteristics of the participants are presented in Table 1.

**Figure 2:**
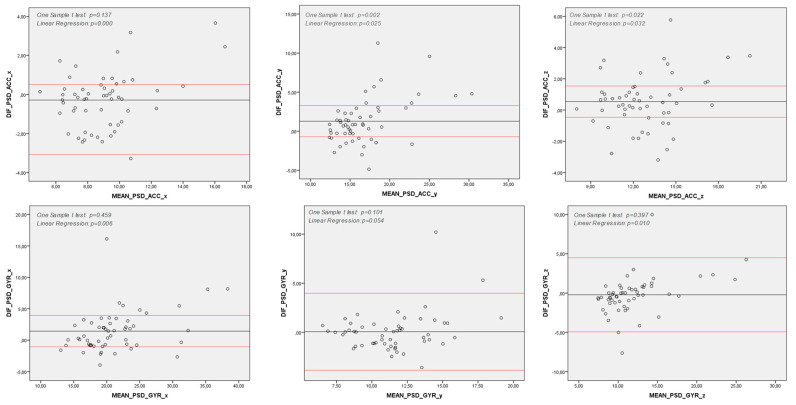
Bland–Altman Analysis of Spectral Variables. *y* axis: Difference between measures (Commercial IMU and Smart Watch) (Hz); *x* axle: Mean between measures (Commercial IMU and Smart Watch) (Hz); Note: DIF: Difference between measures (Commercial IMU and Smart Watch); PSD: Power Spectral Density; ACC: Acceleration (degrees per second); GYR: Angular Velocity (degrees per second); *x*, *y* and *z*: pitch, yaw and roll axes; Note 2: One Sample T-Test *p* < 0.05: differences between Commercial IMU and Smart Watch are significantly higher than zero; hence, there is not agreement between those measurement methods; Note 3: Linear Regression *p* < 0.05: differences between Commercial IMU and Smart Watch tend to concentrate either above or below the differences’ mean; hence, there is a proportion bias between those measurement methods.

**Figure 3:**
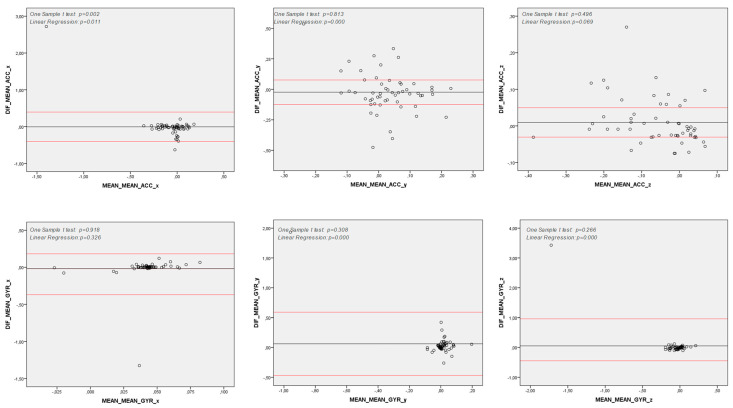
Bland–Altman Analysis of Spatiotemporal Variables. *y* axis: Difference between measures (Commercial IMU and Smart Watch); *x* axle: Mean between measures (Commercial IMU and Smart Watch) Note: DIF: Difference between measures (Commercial IMU and Smart Watch); ACC: Acceleration (degree per second); GYR: Angular Velocity (degree per second); *x*, *y* and *z*: pitch, yaw and roll axes; Note 2: One Sample T-Test *p* < 0.05: differences between Commercial IMU and Smart Watch are significantly higher than zero; hence, there is no agreement between these measurement methods; Note 3: Linear Regression *p* < 0.05: differences between Commercial IMU and Smart Watch tend to concentrate either above or below the differences’ mean; hence, there is a proportional bias between these measurement methods.

The Spearman correlation analysis showed moderate to strong correlations between the G-sensor and smartwatch in spatiotemporal and spectral features for both the accelerometer and gyroscope data (see Table 2). The Bland–Altman analysis demonstrated an agreement between quantitative measures from the G-sensor and smartwatch in almost all features assessed. However, for the spectral features, the differences between the G-sensor and smartwatch were statistically greater than zero in the *x*-axis of acceleration data and the *y* and *z* axes of angular velocity data (see Figure 2). Additionally, a proportional bias was found in all spectral features and mean accelerations in the *x* and *y* axes, indicating that the differences between the G-sensor and smartwatch in these features tended to concentrate either above or below the mean of the differences (Figure 2 and Figure 3).

Table 3 presents the discriminative validity, showing the capability of the evaluation method studied to differentiate PD from healthy control subjects. Statistically significant differences between PD and control subjects were observed in the acceleration data only following the spectral analysis and in the *z*-axis. Angular velocity data, on the other hand, proved capable of differentiating the PD and control groups in angular velocity mean (*z*-axis), angular velocity standard deviation (*y*-axis), and angular velocity power spectral density (*x*-axis).

Concurrent validity, assessed through Spearman correlation, showed moderate to strong correlations between this study’s assessment method and the time since PD diagnosis, the MDS-UPDRS III total score, and rest tremor items from the MDS-UPDRS III (see Table 4).

Reliability analysis of the test was conducted using the Bland−Altman method [41,42] to assess the agreement between two consecutively taken measurements from each subject. Our findings revealed that, for none of the variables studied in the proposed method, the difference between the first and second measurements was significantly greater than zero.

**Figure 4:**
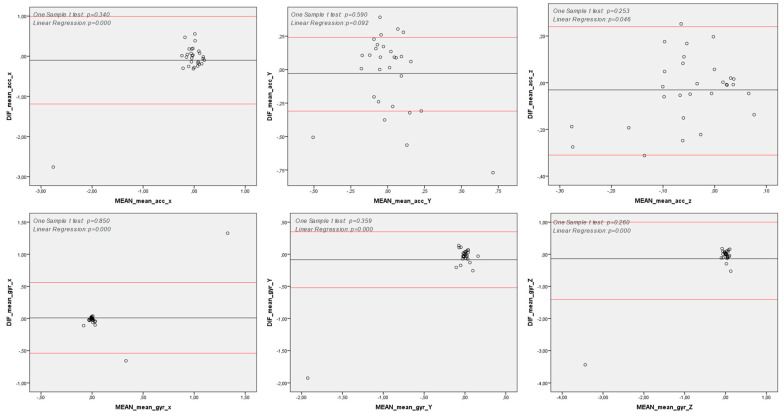
Reliability of Spatiotemporal Variables—Bland-Altman Analysis. *y* axis: Difference between measures (First and second measures); *x* axis: Mean between measures (First and second measures); Note: DIF: Difference between measures (First and second measures); ACC: Acceleration (degrees per second); GYR: Angular Velocity (degrees per second); *x*, *y* and *z*: pitch, yaw and roll axes; Note 2: One Sample T Test *p* < 0.05: differences between first and second measures are significantly higher than zero; hence, there is not agreement between these measurement methods; Note 3: Linear Regression *p* < 0.05: differences between First and second measures tend to concentrate either above or below the differences’ mean; hence, there is a proportional bias between these consecutive measures.

**Figure 5:**
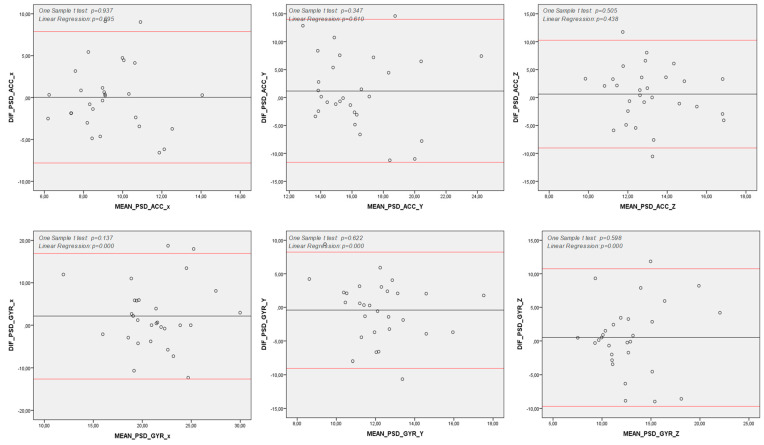
Reliability of Spectral Variables—Bland–Altman Analysis. *y* axis: Difference between measures (First and second measures) (Hz); *x* axis: Mean between measures (First and second measures) (Hz); Note: DIF: Difference between measures (First and second measures); ACC: Acceleration (degrees per second); GYR: Angular Velocity (degree per second); *x*, *y* and *z*: pitch, yaw and roll axes; Note 2: One Sample T Test *p* < 0.05: differences between First and second measures are significantly higher than zero; hence, there is not agreement between those measurement methods; Note 3: Linear Regression *p* < 0.05: differences between First and second measures tend to concentrate either above or below the differences’ mean; hence, there is a proportion bias between those consecutive measures.

Moreover, graphical analysis demonstrated a strong agreement between the two measurements. However, some variables exhibited proportional bias, where intra-measurement differences were concentrated either above or below the mean of the differences (Figure 4 and Figure 5).

Table 5 presents the sensitivity and specificity values for assessing the accuracy in detecting the presence of PD symptoms (action and rest tremor, and bradykinesia), as well as distinguishing PD from healthy control subjects. It also includes the corresponding cutoff points for each evaluated outcome. For the differentiation of PD from control subjects, a cutoff point of 19.71 W/Hz was established for the angular velocity power spectral density on the *x*-axis (PSD_gyr_x), demonstrating a sensitivity of 72% and specificity of 71% in effectively distinguishing PD cases from control subjects (AUC = 0.726).

Additionally, we established the following cutoff points for resting and action tremors in the variable of acceleration power spectral density on the *x*-axis: 9.07 for resting tremor and 6.43 for action tremor. These values yielded a sensitivity of 80% and 73%, and a specificity of 62% and 67%, respectively (AUC rest tremor = 0.711; AUC action tremor = 0.760).

## 4. Discussion

Our initial goal was to examine the agreement between measurements obtained from smartwatch sensors and a commercial wearable inertial sensor. Subsequently, we aimed to explore the algorithm’s discriminative validity, concurrent validity, reliability, and accuracy. The use of an algorithm adapted for smartwatch sensors already available on the market offers a low-cost and scalable solution, which could democratize access to motor assessments for PD patients, especially on underserved communities in developing countries.

Our algorithm demonstrated agreement between the data collected from the commercial IMU and the smartwatch. Additionally, our study uncovered strong correlations between smartwatch-derived data and those obtained from the commercial IMU—renowned as a gold standard for accelerations and angular velocities assessment. [37,38,44,45,46] It was observed, however, that variables assessing Power Spectral Density (PSD) did not exhibit the same level of robustness. We hypothesize that subtle discrepancies arising from the sensitivity of the smartwatch sensors may contribute to variations within a variable that considers minor oscillations in the frequency domain of movement. However, the substantial correlation for spectral analysis variables suggests that, despite the potential sensor-related variations, the fundamental spectral characteristics were preserved; underscoring the robustness of the smartwatch data and its alignment with well-established evaluation methods (commercial IMU).

We observed considerable discriminative validity, as evidenced by the algorithm’s ability to differentiate between healthy individuals and those with Parkinson’s disease. Our developed algorithm effectively distinguished between individuals with Parkinson’s disease and control subjects solely utilizing gyroscopic data (that evaluated angular velocities), rather than accelerometer data, highlighting accelerometers’ limitations in detecting rotations around the vertical axis, which can significantly impact upper limb movement analysis, particularly when assessing hand tremors [45,47]. Our study, which involved a sample of mildly affected Parkinson’s patients during their on-phase, showed that spectral analysis was more effective in detecting disparities between Parkinson’s patients and healthy individuals than spatiotemporal analysis. This highlights spectral analysis’s sensitivity to subtle movement deviations and its potential for a more comprehensive assessment of motor abnormalities [15,44,46,48,49,50,51].

The gold standard for motor assessment in Parkinson’s disease is the MDS-UPDRS III, widely utilized by clinicians worldwide [7]. We have demonstrated that the evaluation of arm movement using a smartwatch shows moderate to strong correlations not only with the total MDS-UPDRS score but also with the specific subscores for upper limb assessment within the MDS-UPDRS (especially the spectral analysis of the accelerometer signals on the *x, y*, and *z* axis, and the gyroscope signal on the *z* axis—Especially the spectral analysis of the accelerometer signals (*x*, *y*, and *z* axes) and the gyroscope signal (*z* axis)—which may provide greater sensitivity in detecting subtle nuances in tremor frequency and amplitude). In agreement with the literature, this demonstrates that the greater the motor impairment due to the disease, the more pronounced the alterations found through our assessment method [12,13,14,15,16,17,52]. Additionally, we found correlations between our method and time since diagnosis of the patients, indicating that the longer the diagnosis time, the greater the quantitative alterations in arm movement. These findings underscore the concurrent validity of our test in evaluating motor symptoms of the upper limb in PD patients. However, it’s important to note that our sample consisted of individuals with mild motor impairment, implying that the results could be more significant if extrapolated to patients with greater impairment and evaluated during the off-medication state.

Arm movement data acquisition was subsequently repeated in each individual during the present study. This was performed to assess the test’s reliability, aiming to establish that when the test was repeated, the data remained consistent. Indeed, Bland–Altman analysis confirmed that the differences between acquisitions were not significantly greater than zero, indicating agreement between the two measurements. Although some evaluated variables may exhibit a proportional bias, with differences concentrated either above or below the mean of the differences between measurements, we hypothesize that this may primarily reflect the subject’s learning effects with the proposed task [53]. Therefore, overall, we considered the repeatability of the evaluated test to be satisfactory.

The present method effectively differentiated arm movements in individuals with and without PD. When evaluating the test’s performance in diagnosing the disease, we observed an area under the curve of 0.72 for the variable of PSD on the *x*-axis of gyroscope data. Additionally, we observed satisfactory performance in detecting PD symptoms (rest (AUC = 0.71) and action tremor (AUC = 0.76)) during upper limb movement (of PSD on the *x*-axis of accelerometer data). It is important to note that these variables (PSD on the *x*-axis, whether from accelerometer or gyroscope data) were chosen for this analysis because higher values indicate pathological tremor in the wrist and hand flexor-extensor muscles, which are often observed in patients with PD [12,21,37]. Moreover, spectral analysis represents a more detailed examination of the raw signal, highlighting even minor pathological alterations that simple time-domain evaluation might not detect [15,44,48,49,50,51].

Integrated biosensors have also been the subject of extensive investigation for the assessment of a wide range of diseases [54]. In the context of Parkinson’s disease, this technology has been harnessed for symptom detection and in distinguishing essential tremors from tremors associated with the disease [55]. Therefore, after analyzing the test’s performance, we conclude that despite some inconclusive data, the overall analysis shows that the arm movement analysis using our algorithm and data collected via smartwatches is a relatively reliable method with good performance across all analyzed properties.

The innovation of assessment on symptoms through wearable devices offers a technique that is both easily applicable and widely accessible, significantly improving care for individuals with Parkinson’s disease [12,13,14,15,16,17,18,19,44,56,57,58]. This study is pioneering in developing—and especially in properly validate—a quantitative method for assessing movement using commercially available accelerometers and gyroscopes for the general population. A major limitation in diagnosing and managing Parkinson’s disease is monitoring subtle motor symptoms, which may not manifest during clinical assessments and are difficult for patients to describe. This method provides a practical, easy-to-use alternative for home use, with remote access for clinicians, enabling comprehensive symptom evaluation. Utilizing data from smartwatches eliminates the need for expensive equipment, positively impacting the understanding and treatment of Parkinson’s disease.

However, it is important to acknowledge the limitations of this study. Our sample predominantly consisted of individuals with mild motor impairment, and their assessments were conducted during their “on” states, when the effects of medication typically suppress motor symptoms. This may have reduced the contrast between subjects with and without the disease, potentially hindering the algorithm’s discriminative capacity. In more severe cases or during “off” medication periods, tremor is generally more pronounced and frequent, which could make detection easier. Therefore, although our algorithm showed promising results even under less favorable conditions (i.e., during the “on” state and in mild cases), we hypothesize that its performance may be enhanced in scenarios where tremor is more evident. Nevertheless, further studies involving patients with greater symptom severity and assessments during “off” states are needed to confirm this hypothesis and establish the method’s effectiveness in broader clinical contexts.

In addition to these clinical limitations, certain methodological constraints must also be considered. Although our synchronization method based on signal pattern alignment may introduce some variability, it was intentionally chosen due to its low cost and ease of implementation. This decision aligns with our goal of proposing a practical and accessible assessment strategy that can be applied in both clinical and home environments without the need for external hardware. Moreover, as our method relies on commercially available smartwatches, it is important to acknowledge that sensor characteristics may vary across brands or even between different model generations. Such variability could affect the reproducibility of results. Future studies should consider cross-device comparisons to better establish the broader applicability and generalizability of this approach.

## 5. Conclusions

Our findings suggest that the proposed algorithm can differentiate between individuals with Parkinson’s disease and healthy controls using angular velocity data from arm movements, showing moderate to strong agreement between commercial IMU and smartwatch sensors. The algorithm demonstrated satisfactory discriminative and concurrent validity, as well as good reliability and accuracy for identifying tremor-related features in a clinical context.

These results highlight the potential utility of smartwatches as complementary tools in Parkinson’s disease assessment and monitoring, particularly for frequency-based features derived from power spectral density. However, Bland–Altman analysis revealed proportional bias in some spatiotemporal metrics, especially on the *z*-axis accelerometer data, indicating the need for cautious interpretation of temporal measurements. While smartwatches offer a portable, low-cost, and scalable alternative to conventional sensors, further studies involving individuals with more advanced disease severity and exploring the impact of medication “on” and “off” states are warranted to broaden validation and clinical applicability.

## Figures and Tables

**Table 1:** Demographic characteristics.

	PD	Control
	(*n* = 21)	(*n* = 27)
Male	8 (38.09)	9 (33.3)
Age, years, mean ± SD	66.92 ± 9.0	57.18 ± 8.9
Height, mean ± SD (m)	1.66 ± 0.90	1.66 ± 0.81
Body Mass (kg)	71.51 ± 10.85	73.22 ± 17.75
MoCA Test, mean ± SD (score)	25.85 ± 3.3	24.74 ± 4.3
SAH	13 (46.4)	12 (44.4)
DM	3 (10.7)	1 (3.7)
Time since diagnosis median (min–max), (months)	72 (4–372)	-
MDS-UPDRS III (0–132) median (min–max), (score)	8 (1−25)	-
H&Y modified, frequency (1/1.5/2/2.5/3/4/5)	11/6/1/2/1/0/0	-

Note: PD: Parkinson’s Disease; SAH: Systemic Arterial Hypertension; DM: Diabetes Mellitus; MDS-UPDRS III: Movement Disorder Society—Unified Parkinson’s Disease Rating Scale III; H&Y: Hoehn and Yahr scale *n*: number of participants; SD: standard deviation; max: maximum; min: minimum.

**Table 2:** Agreement Assessment—Commercial IMU (cIMU) and Smartwatch Correlations.

	Acceleration				Angular Velocity			
	G-sensor	SmartWatch	r	*p*	G-Sensor	SmartWatch	r	*p*
Mean								
*x*	−0.070 (−0.3 to 0.2)	−0.065 (−2.7 to 0.2)	0.45 **	0.001	−0.002 (−1.4 to 0.6)	0.015 (−0.1 to 1.3)	0.323 **	0.02
*y*	0.019 (−0.2 to 0.2)	0.042 (−0.5 to 0.3)	-	-	0.027 (−1.1 to 0.2)	−0.033 (−1.9 to 0.1)	-	-
*z*	−0.052 (−0.4 to 0.1)	−0.061 (−0.3 to 0.1)	0.803 ***	0.000	−0.0306 (−0.2 to 0.2)	−0.084(−3.4 to 0.1)	0.782 ***	0.00
*SD*								
*x*	0.427 (0.2 to 0.5)	0.505 (0.3 to 2.8)	0.756 ***	0.000	0.323 (0.2 to 0.)	0.357 (0.26 to 1.48)	0.294 *	0.03
*y*	0.341 (0.2 to 0.4)	0.388 (0.2 to 0.8)	0.429 **	0.001	0.337 (−0.2 to 0.2)	0.390 (0.2 to 2.1)	0.553 **	0.00
*z*	0.361 (0.2 to 0.5)	0.436 (0.2 to 3.7)	0.627 **	0.000	0.361 (0.2 to 0.5)	0.436 (0.2 to 3.7)	0.608 **	0.,00
*PSD*								
*x*	8.833 (5.0 to 17.8)	9.122 (4.9 to 15.4)	0.761 ***	0.000	21.855 (12.2 to 42.4)	20.41 (11.94 to 34.24)	0.767 ***	0.00
*y*	17.322 (11.6 to 32.7)	16.046 (11.9 to 27.9)	0.699 **	0.000	11.516 (6.86 to 20.4)	11.452 (6.1 to 18.41)	0.815 ***	0.00
*z*	13.053 (8.0 to 21.9)	12.514 (7.98 to 18.47)	0.802 ***	0.000	11.82 (6.7 to 28.4)	12.04 (7.7 to 24.1)	0.717 ***	0.00

**Note:** Data expressed as mean and interquartile range [mean (range … to …)]; cIMU: commercial Inertial Measurement Unit. * Weak correlation r < 0.4; ** Moderate correlation, 0.4 < r < 0.7; *** Strong correlation, r > 0.7.

**Table 3:** Discriminant Validity Assessment—PD and Control Comparison.

	G-sensor				Smart Watch			
	PD	Control	*p*	Effect Size	PD	Control	*p*	Effect Size
Mean								
*x*	−0.0103 (−0.0 to 0.0)	0.005 (−0.0 to 0.0)	-	-	0.037 (−0.0–0.1)	−0.007 (−0.0–0.0)	-	-
*y*	0.030 (−0.0 to 0.0)	0.024 (−0.0 to 0.0)	-	-	–0.0785 (−0.2–0.0)	0.012 (−0.0–0.0)	-	-
*z*	−0.0003 (−0.0 to −0.0) *	−0.062 (−0.0 to −0.0)	0.003	0.844	−0.119 (−0.3–0.1) *	–0.047 (0.0–0.0)	0.02	1.033
*SD*								
*x*	0.331 (0.3 to 0.3)	0.315 (0.3 to 0.3)	-	-	0.374 (0.2–0.4)	0.339 (0.3–0.3)	-	-
*y*	0.333 (0.3 to 0.3)	0.341 (0.3 to 0.3)	-	-	0.411 (0.2–0.5) *	0.369 (0.3–0.3)	0.04	0.165
*z*	0.361 (0.3 to 0.3)	0.361 (0.3 to 0.3)	-	-	0.485 (0.2–0.7)	0.384 (0.3–0.4)	-	-
*PSD*								
*x*	23.800 (21.1 to 26.4) *	19.839 (18.1 to 21)	0.01	0.705	22.080 (19.8 to 24.2) *	18.696 (17.4 to 19.9)	0.009	0.750
*y*	12.613 (11.2 to 13.9) *	10.379 (9.4 to 11.2)	0.008	0.776	11.780 (10.7 to 12.7)	11.112 (10.0 to 12.1)	-	-
*z*	13.381 (11.1 to 13.3) *	10.210 (9.3 to 11)	0.04	0.816	12.990 (11.2 to 14.7)	11.068 (10.2 to 11.8)	-	-

Note: Data expressed as mean and interquartile range [mean (range … to …)]; SD: Standard Deviation; PSD: Power Spectral Density; PD: Parkinson’s Disease; * Significant difference from Control Group, *p* < 0.05.

**Table 4:** Concurrent Validity Assessment—Algorithm and Clinical Features Correlations.

		Time Since Diagnosis		UPDRS III Total		UPDRS III—Rest Tremor		UPDRS III—Action Tremor		9HPT—Dexterity	
Acceleration											
		r	*p* value	r	*p* value	r	*p* value	r	*p* value	r	*p* value
Mean											
*x*	–0.065 (−2.7 to 0.2)	-		-		-		-		-	
*y*	0.042 (−0.5 to 0.3)	-		-		-		-		-	
*z*	–0.061 (−0.3 to 0.1)	-		-		-		-		-	
*SD*											
*x*	0.505 (0.3 to 2.8)	-		-		-		-		−0.379 *	0.047
*y*	0.388 (0.2 to 0.8)	-		-		-		-		-	
*z*	0.436 (0.2 to 3.7)	-		-		-		-		-	
*PSD*											
*x*	9.122 (4.9 to 15.4)	0.427 **	0.023	0.606 ***	0.001	0.455 **	0.015	0.400 **	0.040	-	
*y*	16.046 (11.9 to 27.9)	-		-		-		-		-	
z	12.514 (7.98 to 18.47)	0.438 **	0.020	0.560 **	0.002	-		-		-	
Angular Velocity											
		r	*p* value	r	*p* value	r	*p* value	r	*p* value	r	*p* value
Mean											
*x*	0.015 (−0.1 to 1.3)	-		-		-		-		-	
*y*	–0.033 (−1.9 to 0.1)	-		-		0.425 **	0.024	-		-	
*z*	–0.084(−3.4 to 0.1)	–0.390 *	0.040	-		-		-		-	
*SD*											
*x*	0.357 (0.26 to 1.48)	-		-		–0.373 *	0.050	-		-	
*y*	0.390 (0.2 to 2.1)	-		-		-		-		-	
*z*	0.436 (0.2 to 3.7)	-		-		-		-		-	
*PSD*											
*x*	20.41 (11.94 to 34.24)	-		-		-		-		-	
*y*	11.452 (6.1 to 18.41)	-		0.541 **	0.003	-		-		-	
*z*	12.04 (7.7 to 24.1)	-		-		0.500 **	0.007	-		-	

Note: Data expressed as mean and interquartile range [mean (range … to …)]; SD: Standard Deviation; PSD: Power Spectral Density; UPDRS III: Unified Parkinson’s Disease Rating Scale—Motor Examination; UPDRS III—rest tremor: question 3 of UPDRS motor examination regarding rest tremor; UPDRS III—action tremor: question 4 of UPDRS motor examination regarding action tremor; UPDRS III—bradykinesia (finger tapping): question 7 of UPDRS motor examination regarding bradykinesia during finger tapping movement; UPDRS III—bradykinesia (pronosupination): question 8 of UPDRS motor examination regarding bradykinesia during arm pronosupination movement; 9HPT: Nine Hole Peg Test. * Weak correlation r < 0.4; ** Moderate correlation, 0.4 < r < 0.7; *** Strong correlation, r > 0.7. “-” meaning that no significant correlation were found.

**Table 5:** Accuracy assessment.

	AUC	Cutoff Point	Sensitivity	Specificity
PD vs. Control ^#^	0.705	19.35	71%	67%
Rest Tremor ^Φ^	0.711	9.07	80%	62%
Action Tremor ^Φ^	0.760	6.43	96%	67%

**Note:** rest tremor, action tremor: presence of its symptoms verified through video evaluation performed by a trained clinician (acquired at the same time of smartwatch acquisition); PD: Parkinson’s Disease; Control: healthy subjects; AUC: area under the curve. ^#^ Test Variable: Angular Velocity Power Spectral Density (PSD) on “*x*” axis, ^Φ^ Test Variable: Acceleration Power Spectral Density (PSD) on “*x*” axis.

## Data Availability

The data are not publicly available due to privacy restrictions but are available from the corresponding author upon reasonable request.
